# Fast and accurate automated recognition of the dominant cells from fecal images based on Faster R-CNN

**DOI:** 10.1038/s41598-021-89863-4

**Published:** 2021-05-14

**Authors:** Jing Zhang, Xiangzhou Wang, Guangming Ni, Juanxiu Liu, Ruqian Hao, Lin Liu, Yong Liu, Xiaohui Du, Fan Xu

**Affiliations:** 1 MOEMIL Laboratory, School of Optoelectronic Science and Engineering, University of Electronic Science and Technology of China, Chengdu, 610054 Sichuan China; 2 Department of Public Health, Chengdu Medical College, Chengdu, 610500 Sichuan China

**Keywords:** Health care, Medical research

## Abstract

Fecal samples can easily be collected and are representative of a person’s current health state; therefore, the demand for routine fecal examination has increased sharply. However, manual operation may pollute the samples, and low efficiency limits the general examination speed; therefore, automatic analysis is needed. Nevertheless, recognition exhaustion time and accuracy remain major challenges in automatic testing. Here, we introduce a fast and efficient cell-detection algorithm based on the Faster-R-CNN technique: the Resnet-152 convolutional neural network architecture. Additionally, a region proposal network and a network combined with principal component analysis are proposed for cell location and recognition in microscopic images. Our algorithm achieved a mean average precision of 84% and a 723 ms detection time per sample for 40,560 fecal images. Thus, this approach may provide a solid theoretical basis for real-time detection in routine clinical examinations while accelerating the process to satisfy increasing demand.

## Introduction

From a biological perspective, the metabolic process is an important bridge between biological function and structure^1^. In the human digestive process, food or water enters the oral cavity first; after a series of chewing cycles, the content flows through the esophagus into the stomach. Gastric acid and enzymes digest the contents under gastric motility^2^. Several hours later, the contents are delivered through the duodenum to the small intestine and large intestine^3^. Therefore, fecal matter clearly contains abundant biological information^4^, and images of fecal samples may help identify early abnormal matter at the early stage.

The total worldwide population is close to 7.8 billion, and the male to female ratio is approximately 1.02^5^. According to the WHO disease report, the incidence of digestive disease is 20–40%, and the incidence of gynecological disease is 24.94%^6,7^. Clearly, there is abundant demand for routine clinical examination of feces. Furthermore, these kinds of biological samples are widely accepted in diagnosis due to characteristics such as noninvasiveness^8,9^ and representativeness^10^ and their ability to provide disease-related information^11^. However, another challenge is becoming clear: how to overcome the limitations of manual operation, such as bad odor and aseptic, inefficient and tedious operation^12^. Solutions to these problems have become increasingly urgent for routine clinical examination.

At present, the automatic recognition of tangible components such as cells under the microscope applies mainly to machine vision. However, the traditional machine vision method requires the design of complex feature extractors (such as morphological features and texture features), and many images must be preprocessed before training^13,14^. In addition, the training process is inadequate and complex.

The lack of an automatic recognition algorithm for organic components under the microscope seriously restricts the automation of routine stool analysis. Recently, deep learning technology has been successfully used in image classification, object detection and other computer vision tasks^15,16^. Compared with traditional machine learning methods, convolutional neural networks automatically extract image features, simplify and avoid unnecessary image preprocessing, and improve the validity and accuracy of detection^17–19^. Therefore, we introduce an automated cell-detection approach based on a faster region-based convolutional neural network (Faster R-CNN)^20^, which we term principal component analysis (PCA)-based^21^ Faster R-CNN (PCA-Faster R-CNN).

## Methods

### Ethics approval and consent to participate

The Institutional Review Board and Ethics Committee of the Fourth Affiliated Hospital of Nanchang University approved this study (SFYLL-PJ-2015-001). Written informed consent was provided by all participants. All biological samples were anonymized. All methods were carried out in accordance with relevant guidelines and regulations.

### Fecal sample collection

In total, 676 positive samples were collected from the Fourth Affiliated Hospital of Nanchang University. These samples were diluted, stirred, allowed to stand and finally sent to a flow cell. To observe a clear sample image, an OLYMPUS CX31 was used in the optical system as the basic optical structure with a 40 × objective lens [numerical aperture (NA): 0.65, material distance: 0.6 mm]. An EXCCD01400KMA CCD camera was used to capture images with 6.45 µm resolution, and a standard halogen lamp was chosen for illumination. Ten to 15 images were collected from each subject in different visual fields.

The size of the collected images was 1600 × 1200. Annotation of each image was conducted manually as the ground truth. The location and size of (RBCs), white blood cells (WBCs), pyocytes (PYOs), and mildews (Mids) were recorded according to the image analysis. Only the standard cell structure was annotated from the images, and the defocused image was not marked to reduce false detection of impurities. A total of 8785 images with stylized components were collected. Training a on a small number of images can affect the test performance of a model. Therefore, to reduce the effect of overfitting, data augmentation was performed using random vertical and horizontal flipping and random contrast and saturation adjustments.

### Proposal

**Figure 1:**
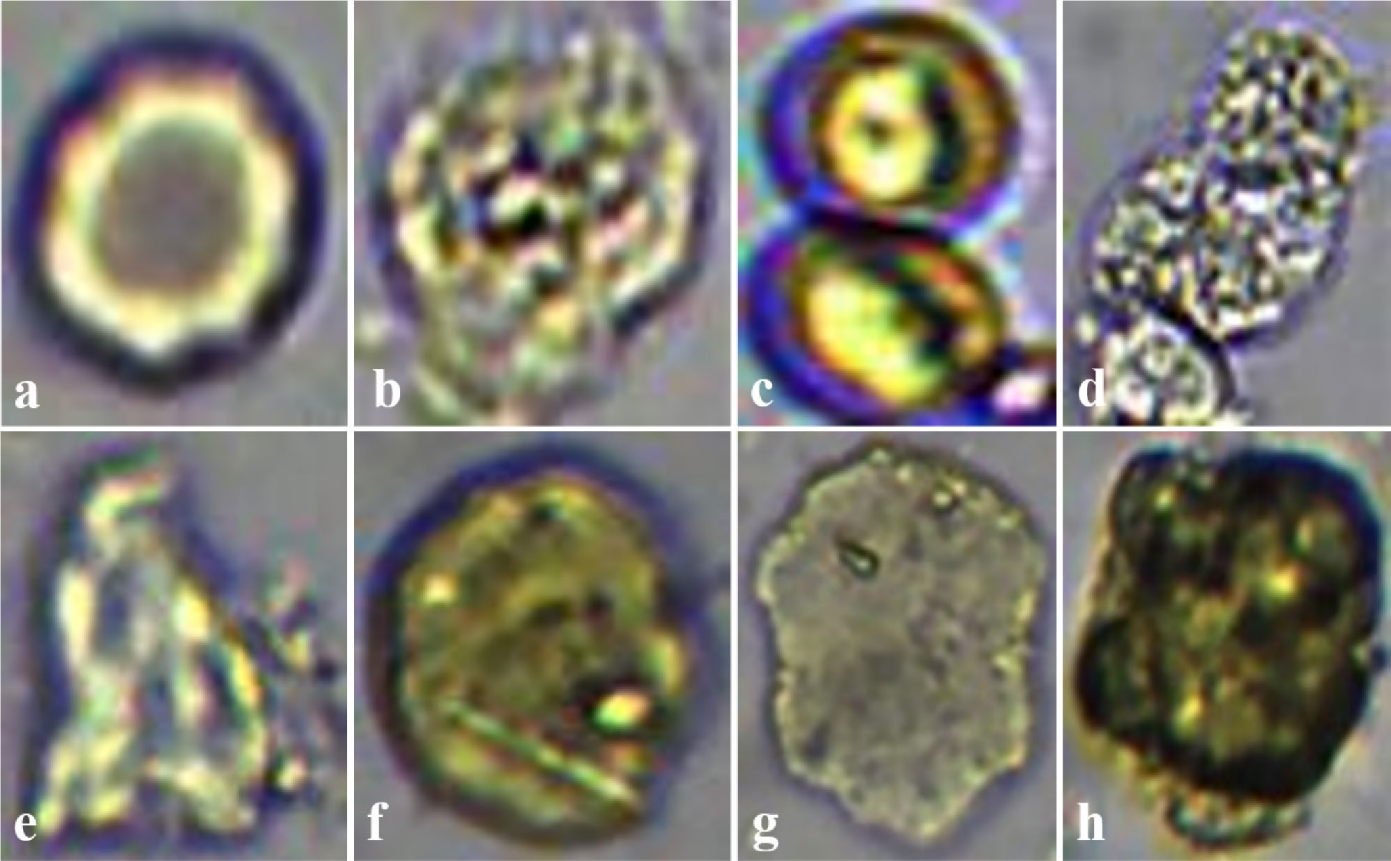
Cells and impurities in fecal samples. (**a**) RBC, first layer with a black outer cycle, second layer with a highlighted irregular cycle, inner with brown cycle; (**b**) WBC, generally round, with noise like the texture inside; (**c**) Mid, budding mold is generally composed of multiple spherical joints; (**d**) PYO, usually formed by adhesion of multiple leukocytes; (**e**,**f**) are different impurities; (**h**) impurity.

Four main elements must be identified during routine fecal examination: RBCs, WBCs, PYOs, and Mids. Other components, such as calcium oxalate crystals, starch granules, pollen, plant cells, plant fibers and food residues, are classified as impurities with less clinical significance. For details, please see Fig. 1a–h.

**Figure 2:**
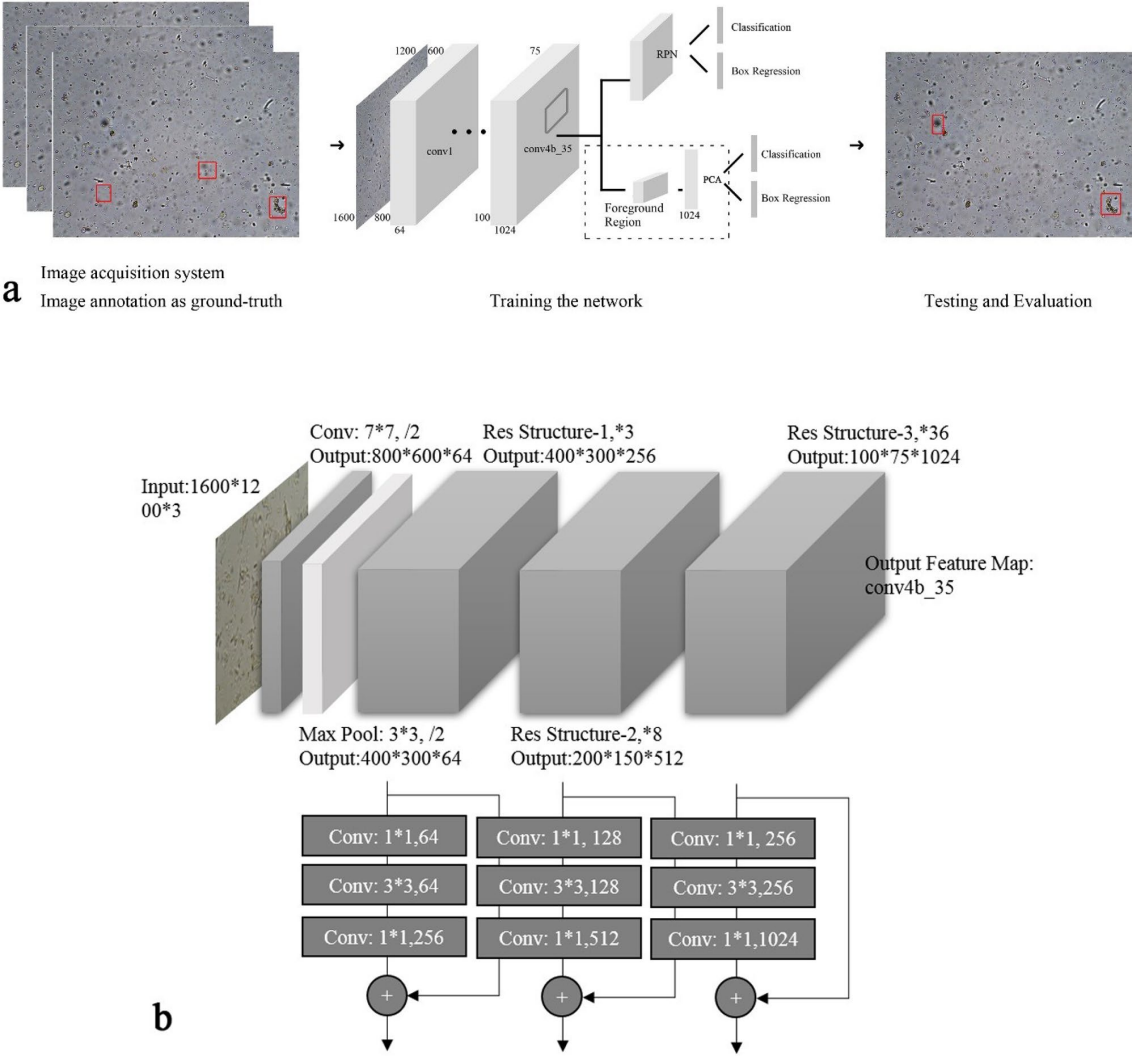
Overall workflow of the proposed approach and sharable 143 CNN layers of ResNet-152. (**a**) Image acquisition system. (**b**) Output feature map. Drawn by DXH.

Faster R-CNN^20^ consists of three main parts: (1) a feature extraction layer, (2) a region proposal network (RPN), and (3) a classification and regression network; see Fig. 2 for a detailed model schematic diagram. Among them, the RPN and classification and regression network share the previous feature extraction layer, as shown in Fig. 2a. The feature extraction layer is composed of a series of convolutional neural networks composed of a convolutional layer, pooling layer, and activation layer. According to the feature map generated by the feature extraction layer, the RPN can generate anchors of different sizes and aspects, which are then used to generate the region proposal. The proposed region generated by RPN is input into the classification and regression network for the type recognition and box accurate regression. Because the scale of the feature map layer corresponding to different foreground regions is inconsistent, Fast R-CNN adopts a region of interest (ROI) pooling strategy to unify the dimensions. Although the calculation is simplified, some features are lost; therefore, we propose PCA dimension reduction to normalize the dimensions of the features.

The feature extraction layers use Resnet^20^, a 152-layer network composed of four residual network blocks: the first three residual network blocks are selected as feature extractors (see Fig. 2b).

**Figure 3:**
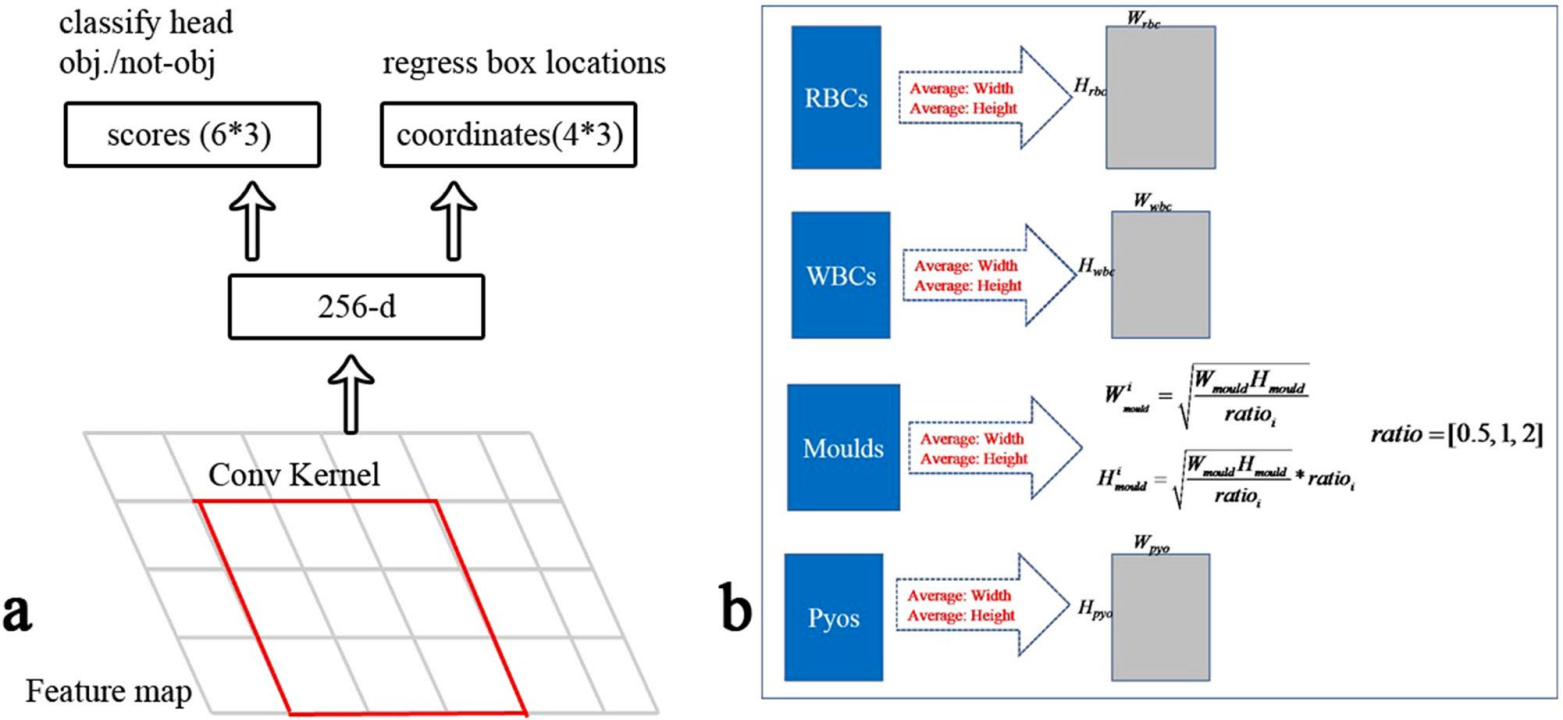
(**a**) Architecture of RPN; (**b**) Generation of anchors. Drawn by DXH.

The RPN was used to generate a batch of proposals, similar to the selective search used in R-CNN^22^ and Fast R-CNN^23^. The network structure is consistent with the RPN used in Faster R-CNN: a 256-channel output is generated by a 3 × 3 convolutional layer after the feature map layer (conv4b_35), which is used to fuse the information around the features and to fuse information across channels. Meanwhile, the fused layer is connected by two branches, termed the SoftMax classification head and box location regression head; for details, see Fig. 3a. In contrast to the RPN in Faster R-CNN, whose box dimensions are hand selected, the generated anchors are based on the average size of the foreground target, which allows the regression network to run smoothly to learn and predict good locations; for details, see Fig. 3b.

**Figure 4:**
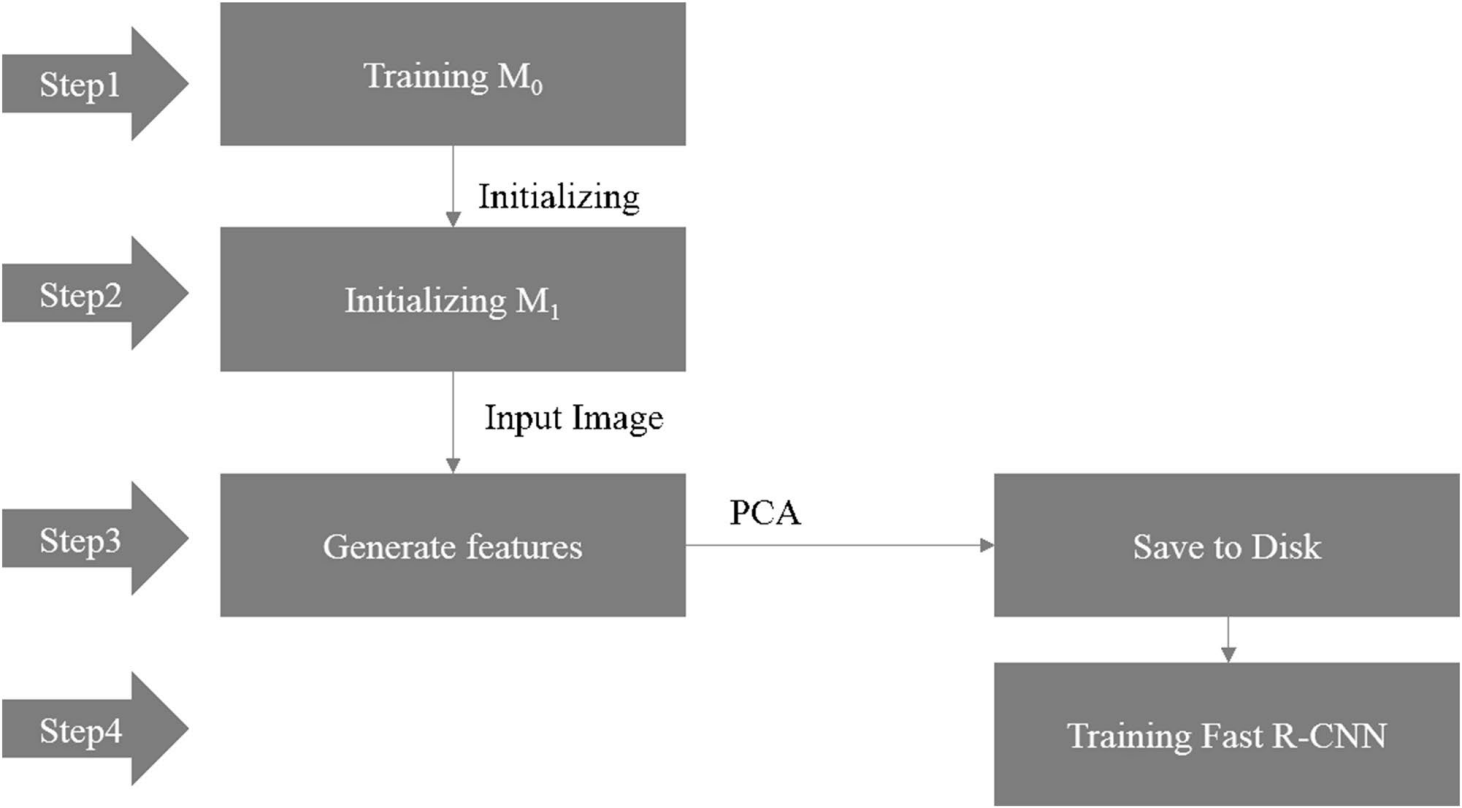
Training process of the PCA-based Faster R-CNN. Drawn by DXH.

In the training process, the RPN module is trained jointly, rather than alternately, with the object recognition network. Since the structure of the Faster R-CNN is end to end, both the RPN and the object recognition network can provide feedback on the feature extraction layer. During backpropagation, the loss functions from both the RPN and the Fast R-CNN are combined and calculated together. Moreover, we introduce the PCA strategy in the classification and regression component of Faster R-CNN that should be trained separately. The original Faster R-CNN model, denoted by M0, can improve the RPN network (3.1.2) and the ROI pooling strategy. PCA-based Faster R-CNN is denoted by M1. The training process is shown in Fig. 4.

### Experimental setup

All experiments were conducted using models developed based on TensorFlow^24^, which provides libraries to build the main structure of deep learning models. The experiments were executed on a Windows system with an Intel Core i7-5960X CPU @ 3.0 GHz × 8, an NVIDIA GeForce GTX 1080 Ti GPU and 32 GB of RAM. The microscopy process involved taking five images with different focal lengths and recording 12 fields of view by means of a movable platform.

## Results

In total, 676 biological samples were obtained from the Fourth Affiliated Hospital of Nanchang University. Therefore, 40,560 fecal images were used to develop the detection algorithm based on Faster R-CNN. All images were collected independently from the microscopic imaging system. The best resolutions of the 12 images were collected for each sample. To further validate the algorithm, experienced laboratory experts annotated the cells of all images in the development dataset with different colors of rectangular boxes as the ground truth. For more details, please see Supplementary Information S1. Detailed fecal sample information and the dataset split are summarized in Table 1.

**Table 1:** Overview of the dataset.

Contents	Dataset A: training	Dataset B: validation	Dataset C: testing
# images	6150	880	1755
Cells	12,348	388	2628
RBCs	5588	1	1572
WBCs	682	5	454
PYOs	70	0	54
Mids	6008	382	548

The dataset is divided randomly. As some samples are negative, they contain fewer cells.

**Figure 5:**
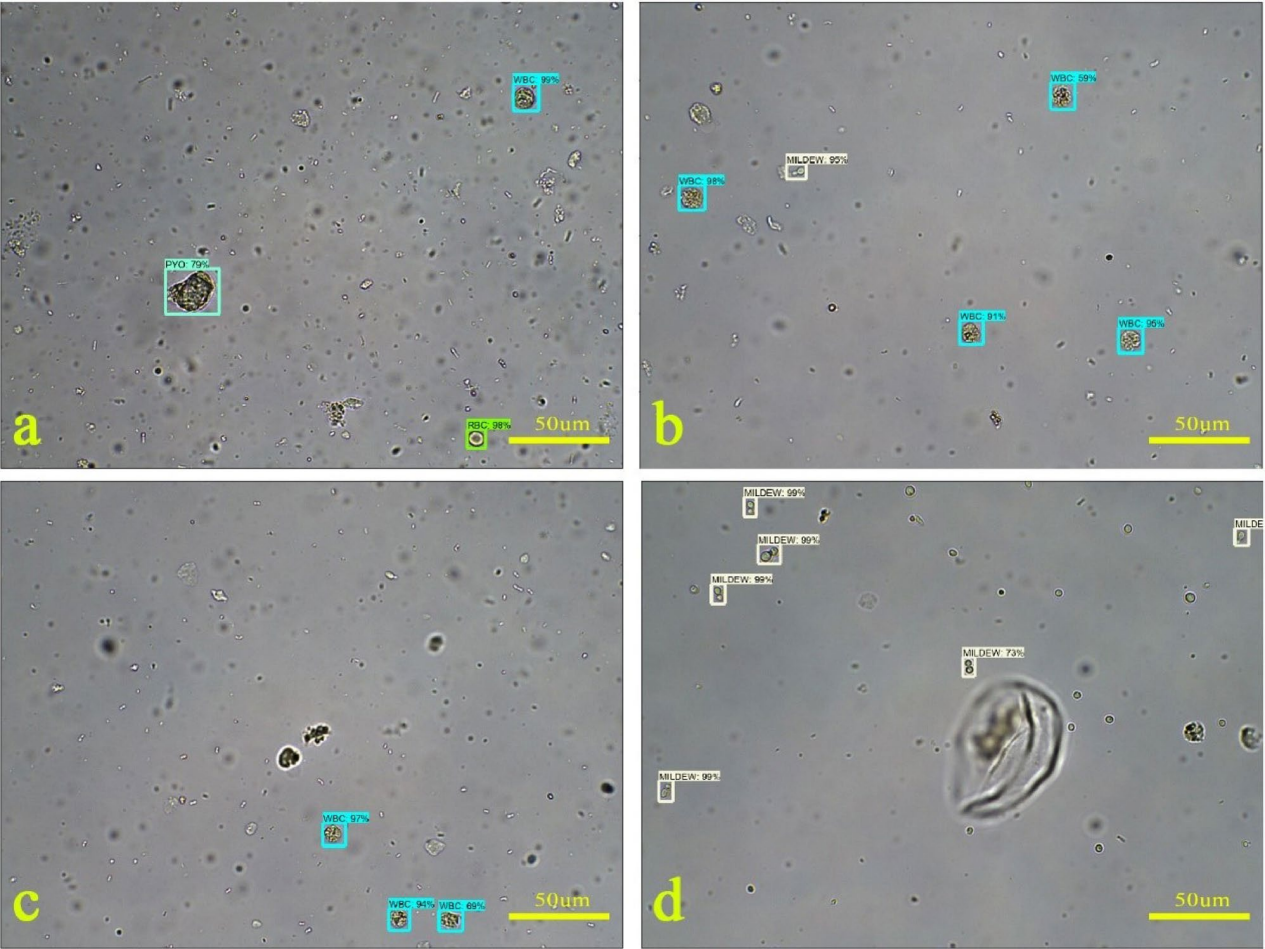
Curated examples of this model on our dataset. A score threshold of 0.6 is used for display. (**a**) PYO, WBC and RBC; (**b**) WBC, Mid, (**c**) WBC, (**d**) Mid.

After training, the network was tested. The WBCs are marked with blue squares and percentages (Fig. 5a–c), while the RBCs are marked with green squares and percentages (Fig. 5a). PYOs are marked with light blue squares and percentages (Fig. 5a). Furthermore, the remaining components, Mids, are marked with gray squares and percentages (Fig. 5b,d); for details, please see Fig. 5.

Average precision (AP) and mean average precision (mAP) were used to detect the cells and identify their location from the microscopic images. Due to the insufficient sample size during training, the detection recognition rate was low. For example, for RBCs, WBCs and Mids, the detection results reflected the performance of the model, and the *m*AP value was 84%. Two established classes of methods are used for object detection in images: one based on morphology segmentation or selective search, which is used in R-CNN and Fast R-CNN, and the other based on region proposal classification. We compared the proposed model of PCA-based Faster R-CNN with R-CNN, Fast R-CNN, Faster R-CNN and R-FCN^25^. The mAP of our method was the highest (0.84). Moreover, the time consumed per image (723 ms) was significantly shorter than that of R-CNN and Fast R-CNN, whereas no significant difference was observed with respect to Faster R-CNN and R-FCN. Specifically, the AP values for RBCs, WBCs, PYOs and Mids were 0.92, 0.85, 0.81, and 0.75, respectively. The AP was 0.84; moreover, the AP values for the four types of cells obtained with our proposed method were higher than those of the other four methods (see Table 2).

**Table 2:** Comparison of 5 cell-detection algorithms.

Model	mAP	Dur/image	AP			
			RBC	WBC	Mid	PYO
R-CNN	0.64	14.9 s	0.80	0.74	0.77	0.26
Fast R-CNN	0.66	4.2 s	0.81	0.77	0.79	0.28
Faster R-CNN	0.80	517 ms	0.89	0.81	0.78	0.72
R-FCN	0.81	468 ms	0.90	0.80	0.80	0.73
SSD300	0.63	78 ms	0.475	0.615	0.621	0.821
SSD512	0.74	123 ms	0.630	0.752	0.654	0.812
YOLOv3	0.58	149 ms	0.548	0.628	0.655	0.486
Cascade R-CNN	0.69	263 ms	0.551	0.629	0.775	0.815
PCA-Faster-R-CNN	0.84	723 ms	0.92	0.85	0.81	0.78

Dataset C was used to validate the average precision.

Clearly, the selective search segmentation method used by R-CNN and Fast R-CNN consumed substantial time. With the introduction of PCA into the feature extraction layer, the features were assigned the main component during the classification and regression process, and the features of Faster R-CNN and R-FCN were filtered out through the pooling strategy. These results also indicate that the Faster R-CNN method based on PCA had the highest overall recognition rate.

**Figure 6:**
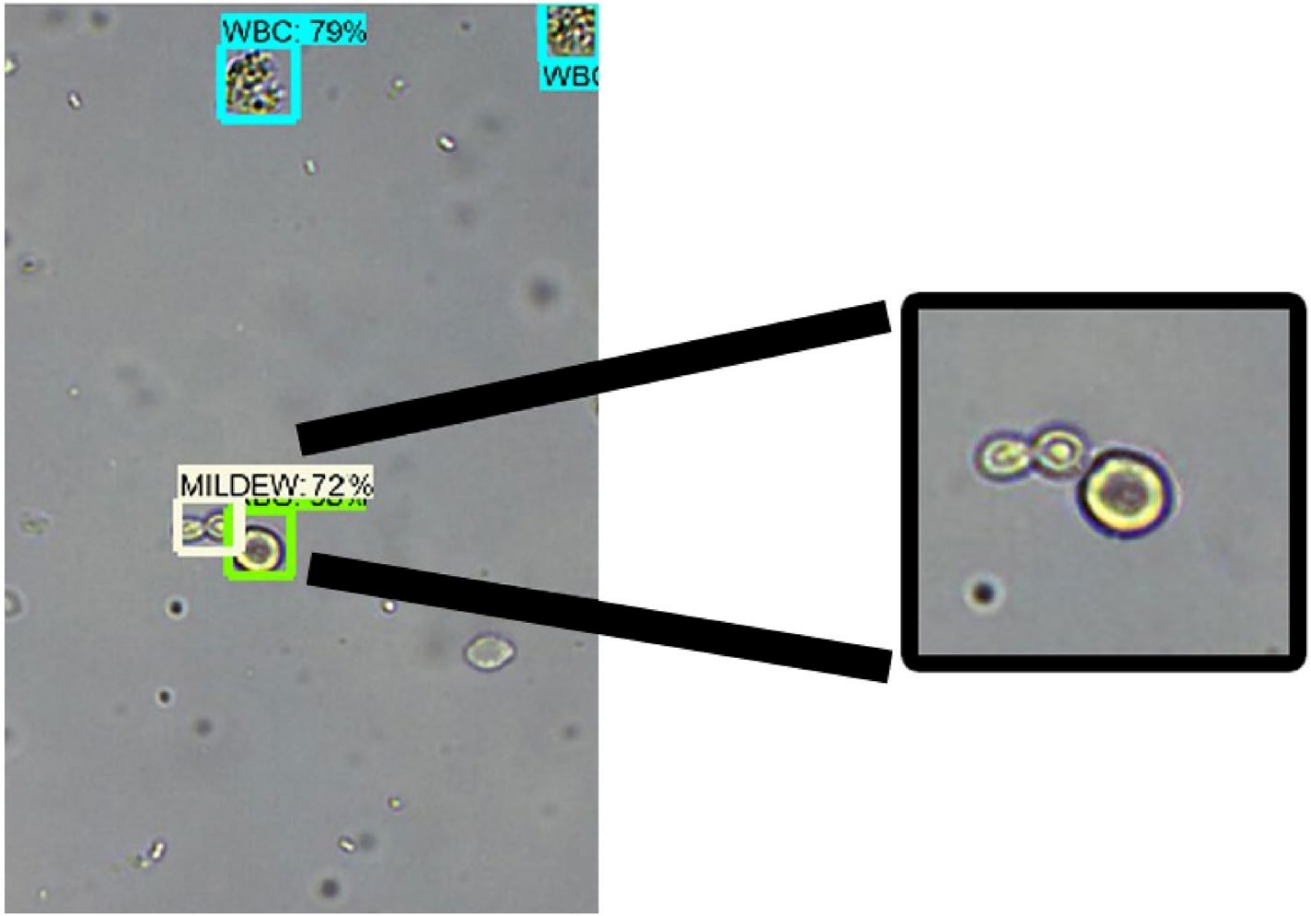
Detection result for adhesion example.

The large number of impurities in the fecal samples made the background of the images complex. Inevitably, the pattern components in the images were difficult to address. However, our algorithm can effectively distinguish the adhesive type components. Unfortunately, the morphological or selective search method cannot accomplish this task. For instance, when an RBC and Mid in the image were stuck together, our algorithm could distinguish the two components (see Fig. 6).

## Discussion

In summary, 676 fecal samples and 40,560 microscopic images were prepared for algorithm development. Our algorithm presented good performance in identifying four kinds of cells and their locations in microscopic images. The algorithm has two major advantages, the average time required to analyze a sample and accuracy.

Clearly, our algorithm consumes significantly less time than R-CNN and Fast R-CNN, which may be due to the introduction of RPN. The R-CNN and Fast R-CNN models use selective search in the segmentation of foreground objects, which requires considerable running time. Each foreground target unit propagates forward to extract features in R-CNN post segmentation^22^, while Fast R-CNN shares the convolutional layer, which can extract features by propagating forward once^23^. However, no significant exhaustion time difference was found between Faster R-CNN and R-FCN. R-FCN uses the position-sensitive map method to avoid the fully connected layer and simplify the training parameters; consequently, the time consumption is slightly lower than that of Faster R-CNN. The time consumption of PCA-Faster-R-CNN is slightly higher, mainly because of the introduction of the PCA strategy after feature extraction.

With respect to the AP performance for four kinds of cells from a single image, the AP of RBCs was the best (0.92), which we believe to be a result of the obvious characteristics of RBCs and the fact that there are no significant morphological changes for different RBCs. The number of RBCs in the collected dataset is large, and data enhancement is adopted to improve the training of RBCs.

The AP values of WBCs and Mildew were 0.85 and 0.81, respectively. This reduced performance may be caused by the specific characteristics of cells in different views. In different samples, leukocytes may be round and influenced by osmotic pressure or be shaped as irregular ellipses. Similarly, different Mids have different spore numbers, sizes and shapes after budding, so the recognition rate is lower than that of RBCs. Meanwhile, due to the sample size, the accuracy of Mids is slightly better than that of WBCs. Furthermore, the AP of PYOs was 0.78, likely a result of the small sample size and insufficient training. PYOs are usually composed of many WBCs with large irregular shapes. Due to the small sample size, the training model suffered from a certain degree of overfitting.

Notable, our algorithm presented better mAP (0.84) than the other methods. The results indicate that PCA plays an important role in feature selection. After introducing PCA into our algorithm model, we proposed a model training method that did not follow the end-to-end architecture of the original Faster R-CNN. The disadvantage is that the model did not represent imbalanced samples well. For example, the number of PYOs was small, and the AP was relatively low compared with that of other types of cells. The PCA-Faster-R-CNN model can be used in other fields of recognition of components in microscopic images, such as target detection in leucorrhea, type component detection in urine, and cell counting in blood.

## Conclusion

A deep learning model for cell detection is proposed for locating and identifying objects from microscopy images. The algorithm achieves the highest *m*AP and has the ability to detect and locate RBCs, WBCs, Mids, and PYOs rapidly. The *m*AP is approximately 84%, and the detection time is 723 ms per image (1600 × 1200 resolution).

### Limitation

Due to the small sample size in the collected dataset, fat globules were not considered in this analysis. When the number of samples belonging to a certain category is small—for example, PYOs—as training proceeds, the model can easily suffer from overfitting. Artificial adhesion of leukocytes can be used to expand the number of samples via data enhancement.

## Supplementary Information

Supplementary Information.

## References

[1] Langemann D, Rehberg M (2010). Unbuffered and buffered supply chains in human metabolism. J. Biol. Phys..

[2] Zorn AM (2017). Development of the digestive system. Semin. Cell Dev. Biol..

[3] Friedman, J. E. T. M. H. F. *The Normal Physiology of the Digestive System*. 1–65 (Heidelberg, 1961).

[4] Obokhare I (2012). Fecal impaction: A cause for concern?. Clin. Colon Rectal Surg..

[5] *World population*. (Accessed 22 December 2020); https://countrymeters.info/en/World.

[6] Dossett ML, Cohen EM, Cohen J (2017). Integrative medicine for gastrointestinal disease. Prim. Care.

[7] Ji N (2018). Disease burden for gynecological disease in China. Zhonghua Fu Chan Ke Za Zhi.

[8] Gerber PF, Opriessnig T (2015). Detection of immunoglobulin (Ig) A antibodies against porcine epidemic diarrhea virus (PEDV) in fecal and serum samples. MethodsX.

[9] Rezasoltani S (2019). The gut microflora assay in patients with colorectal cancer: In feces or tissue samples?. Iran J. Microbiol..

[10] Martinez-Guryn K, Leone V, Chang EB (2019). Regional diversity of the gastrointestinal microbiome. Cell Host Microbe.

[11] Kim HK, Kostidis S, Choi YH (2018). NMR analysis of fecal samples. Methods Mol. Biol..

[12] Abraham BP (2018). Fecal lactoferrin testing. Gastroenterol. Hepatol. (N. Y.).

[13] Manik, S., Saini, L. M. & Vadera, N. In *2016 IEEE 1st International Conference on Power Electronics, Intelligent Control and Energy Systems (ICPEICES).* 1–5.

[14] Ghosh P, Bhattacharjee D, Nasipuri M (2016). Blood smear analyzer for white blood cell counting: A hybrid microscopic image analyzing technique. Appl. Soft Comput..

[15] Afridi MJ (2017). Intelligent and automatic in vivo detection and quantification of transplanted cells in MRI. Magn. Reson. Med..

[16] Zhang J (2017). Computerized detection of leukocytes in microscopic leukorrhea images. Med. Phys..

[17] Simonyan, K. & Zisserman, A. Very deep convolutional networks for large-scale image recognition. arXiv 1409.1556 (2014).

[18] Szegedy, C. *et al.* In *2015 IEEE Conference on Computer Vision and Pattern Recognition (CVPR).* 1–9.

[19] Salvi M, Acharya UR, Molinari F, Meiburger KM (2021). The impact of pre- and post-image processing techniques on deep learning frameworks: A comprehensive review for digital pathology image analysis. Comput. Biol. Med..

[20] Ren S, He K, Girshick R, Sun J (2017). Faster R-CNN: Towards real-time object detection with region proposal networks. IEEE Trans. Pattern Anal. Mach. Intell..

[21] Jolliffe IT (1986). Principal Component Analysis.

[22] Girshick, R., Donahue, J., Darrell, T. & Malik, J. In *2014 IEEE Conference on Computer Vision and Pattern Recognition.* 580–587.

[23] Girshick, R. In *2015 IEEE International Conference on Computer Vision (ICCV).* 1440–1448.

[24] *Tensorflow*. (Accessed 22 December 2020); https://tensorflow.google.cn/.

[25] Dai, J., Li, Y., He, K. & Sun, J. In *Proceedings of the 30th International Conference on Neural Information Processing Systems* 379–387 (Curran Associates Inc., 2016).

