# Supplementary material for: Fast and accurate automated recognition of the dominant cells from fecal images based on Faster R-CNN
Source: Sci Rep. 2021 May 14;11:10361. doi: 10.1038/s41598-021-89863-4 (PMC8121882; doi:10.1038/s41598-021-89863-4)
Supplement: Supplementary file 1 — Supplementary Information. [file 41598_2021_89863_MOESM1_ESM.pdf]

Fast and accurate automated recognition of the dominant cells from fecal images based on Faster R-CNN

Jing Zhang[1], Xiangzhou Wang[1], Guangming Ni[1] Juanxiu Liu[1], Ruqian Hao[1], Lin Liu[1], Yong Liu[1], Xiaohui Du[1,*] , Fan XU[2*]

1. MOEMIL Laboratory, School of Optoelectronic Science and Engineering, University of Electronic Science and Technology of China, Chengdu, China, 610054
2. Department of Public Health, Chengdu Medical College, Sichuan 610500

Correspondent author:

Dr Xiaohui Du

MOEMIL Laboratory

Optoelectronic science and engineering

University of electronic science and technology of China

Chengdu, Sichuan, China 610054

Email: xiaohuie@126.com

Prof FAN XU

Department of Public Health

Chengdu medical college

Chengdu, Sichuan

China, 610500

Email: xufan@cmc.edu.cn

Supplementary document

Conventional computer vision techniques for cell detection

With the extensive development and application of visual detection technology of microscopy, many detection images will be generated during the detection process. Manual interpretation is required to obtain test results, which are inefficient and ineffective. Computer vision is the ability of a computer or machine to acquire human-like understanding from digital images or videos[1]. Generally, applications of conventional computer vision methods include image pre-processing, image segmentation, feature extraction, object recognition and structural analysis[2,3].

Machine learning recognition based on the targets' morphology is one of the most conventional detection methods. For example, Piuri and Scotti[4] use a method of comparing stretch and open operation structure elements to extract the characteristics of white blood cells. Finally, Basophil, Eosinophil, Lymphocyte were classified by neural network. Wang and Zhou[5] utilize both the intensity and shape information of the cell for better segmentation quality. In their paper, an online support vector classifier (OSVC) is used for cell phase identification. The segmentation method of intensity maxima was adopted by Shubham Manik[6], and 8 morphological features of white blood cell binary images were extracted, classified and identified by ANN. In addition, there are machine learning recognition methods based on cell texture features. Ghosh[7] separate the individual regions, then, those regions are classified by the shape, size, color and texture features independently with different fuzzy and non-fuzzy techniques.

However, a major limitation of the conventional computer vision technology applied in the above studies is the need to design complex feature extractors suitable only for specific tasks. Another problem is that a lot of image preprocessing is required to prepare the training data set, and the training process is tedious. As the morphological method is usually used to obtain the foreground objects, it is easy to miss and over segment in the microscopic images with complex background such as fecel. In addition, previous studies mainly focused on the recognition and property retrieval of single cell types, and few studies on automatic recognition and localization of other common cells, such as RBC, WBC, mildew.

Deep learning-based approaches for image classification and object detection

The essence of deep learning is to build a machine learning model with many hidden layers and massive training data to learn more useful features, thus ultimately improving the accuracy of classification or prediction. Different from the traditional shallow learning, it emphasizes the depth of the model structure, usually with 5, 6, or even more layers of hidden nodes and the importance of feature learning is clearly highlighted. In other words, the feature representation of the sample in the original space is transformed to a new feature space through layer by layer feature transformation, so as to make classification or prediction easier. Compared with the method of artificial rule construction, using big data to learn features is more able to depict rich internal information of data.

Currently, there were some studies attempting to apply deep learning approaches for cell detection. Edward Kim[8] et al. used the fine-tuning AlexNet model for thyroid cytopathology classification between follicular cells to malignant tumors. Afridi[9] et al. use the proposed deep convolutional neural network (CNN) architecture for automated spot detection in MRI data, an in vivo accuracy up to 97.3% and in vitro accuracy of up to 99.8% is achieved. Jing[10] et al. take the SVM model and CNN architecture to classify the leukocytes in microscopic images after the segmentation. Our previous studies[11] extract the HOG features from the CNN feature maps and classify the fungi in microscopic leucorrhea images with the SVM model after the PCA.

However, the application of CNNs for infrastructure inspection is still in its infancy and there has been limited research using CNNs for multi-object cell detection. In addition to the various cell types examined, the cell localization information is also desired. Region proposal convolutional neural network (R-CNN)[12] is one of the typical deep learning methods to detect the cells. The shortcoming of R-CNN is that it is slow for each input, and the features of proposals forward propagation for each region are needed. The other one is SVM and box regression are not in the same pipeline with CNN, therefore, SVM and box regression cannot respond to CNN features. To solve the problem of large calculation of R-CNN, Fast R-CNN[13] proposed two improvement schemes. Firstly, R-CNN needs to calculate the features of forward propagation for each region proposal, while Fast R-CNN shares the convolution layer before the region proposal. Secondly, Fast R-CNN implements end-to-end training, that is, classification uses full-connected layer to replace SVM and shares a pipeline with box regression.

However, Fast R-CNN still relies on Selective Search to produce proposals, which greatly affects the efficiency of detection. Faster R-CNN[14] adopts another idea to

produce the proposal, namely region proposal network (RPN). Thus, Faster R-CNN consists of two different networks, one is RPN and the other Fast R-CNN detector, which is used for classification and regression. The two networks share the convolution layer to extract features, which greatly simplifies the calculation quantity and the speed. Meanwhile, the detection accuracy rate is greatly improved compared with R-CNN and Fast R-CNN because of RPN.

Zhang J[15] combines Faster R-CNN with the proposed CSA which can effectively detect and identify detection and identification of cancer cells. Jane Hung[16] use the Faster R-CNN to detect malaria parasites in bright field microscopy images of malaria-infected blood. Rui Kang[17] treat the urine particles recognition as object detection and exploit two state-of-the-art CNN-based object detection methods, Faster R-CNN and SSD, as well as their variants for urine particles recognition. To our best knowledge, there is no study using the Faster R-CNN for detecting the cells in fecal microscopic images.

There are four main formed elements in fecal routine examination that need to be identified: red blood cells, white blood cells, pyocytes, and mildews. Other components such as calcium oxalate crystals, starch granules, pollens, plant cells, plant fibers and food residues are all impurities and have no clinical significance. As shown in Figure 1, (a)~(h) are different types of formed elements in fecal samples.

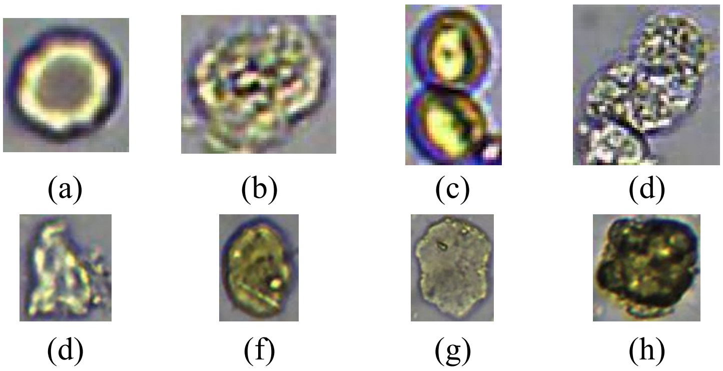

Figure 1. Cells and Impurities in fecal samples. **(a)** RBC; **(b)** WBC; **(c)** mildew; **(d)** pyocyte; **(e)~(f)** are different impurities; **(h)** impurity;

3. The proposed method for cell location and classification

Compared with other target detection algorithms, Faster R-CNN has higher detection accuracy rate and regression rate on the ImageNet[18] data set. The model schematic diagram presented in this paper is shown in Figure 2.

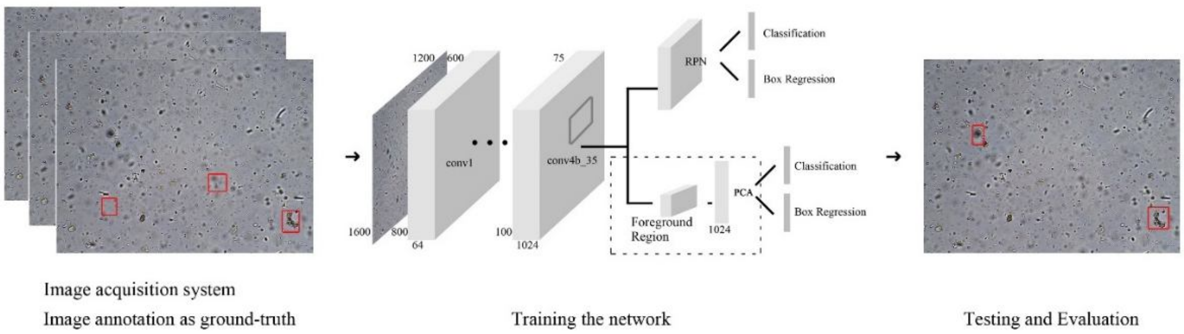

Figure 2. Overall workflow of the proposed approach

3.1 PCA Faster R-CNN model construction

Faster R-CNN mainly consists of three parts: feature extraction layer, region proposal network (RPN), classification and regression network. Among them, the RPN, classification and regression network share the previous feature extraction layer, as shown in Figure 2. Feature extraction layer is composed of a series of convolutional neural networks, such as convolution layer, pooling layer, activation layer, etc. According to the feature map generated by feature extraction layer, the RPN can generate anchors of different sizes and aspects, which are used to generate the region proposal. The proposed region generated by RPN is input into the classification and regression network for the type recognition and box accurate regression. Because the scale of the feature map layer corresponding to different foreground regions is inconsistent, Fast R-CNN adopts ROI Pooling strategy to unify the dimensions. Although the calculation is simplified, some features are lost to some extent. Therefore, we propose to use PCA dimensional reduction method to normalize the dimensions of features. The detailed steps are as follows.

3.1.1 Feature extraction layers

The main architecture of Faster R-CNN consists of Resnet -152[19], as depth residual network shows high precision and recall on ImageNet[18] and COCO[20] datasets. Resnet is a 152-layer network composed of 4 residual network blocks. Among them, the first three residual network blocks are selected as feature extractors, as shown in Figure 3.

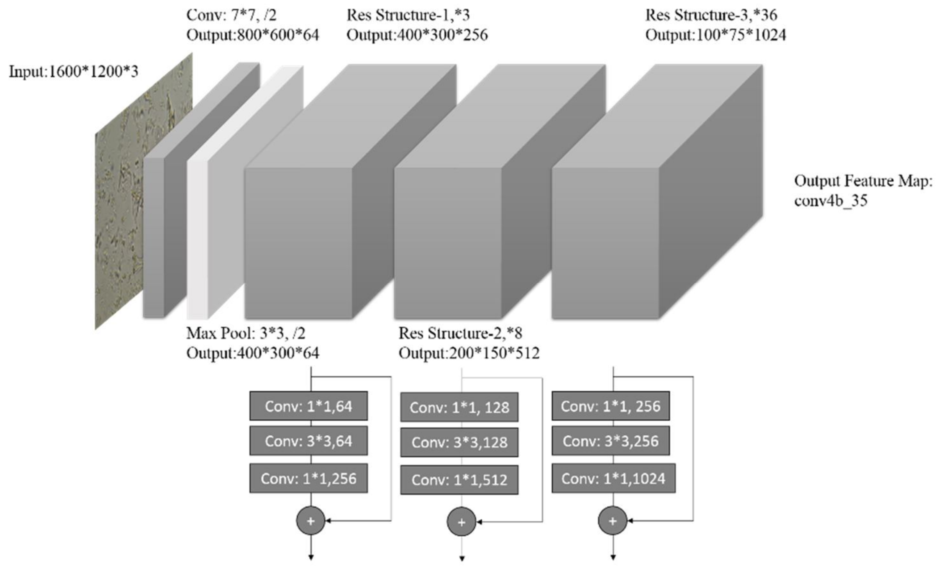

Figure 3. Sharable 143 CNN layers of Resnet-152

Initially, the input image size is 1600 * 1200, and the color image with 3 channels is first processed by a 7 * 7 convolution layer (stride: 2, channels: 64) and a 3 * 3 maximal pooling layer (stride: 2). Then, three different residual structures are connected, and the final output characteristic image (conv4b_35) size is 100 * 75 * 1024.

3.1.2 Region proposal network (RPN) for cell detection

The RPN network is used to generate a batch of proposals which is like the Selective Search used in R-CNN and Fast R-CNN. The network structure is consistent with the RPN used in Faster R-CNN: a 256-channel output is generated by a 3 * 3 convolution layer after the feature map layer (that is, conv4b_35), which is used to fuse the information around the features and to fuse information across the channels, and the fused layer is connected by two branches, which called softmax classification head and box location regression head, as shown in Figure 4.

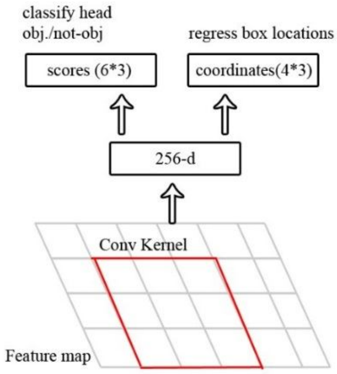

Figure 4. Architecture of RPN

In the process of producing anchor, taking into account the smaller size of the formed elements (RBC size:40~60, WBC size: 60~80, mildew size:30~108, pyocyte size: 90~130) in microscopic image and the larger width (or height) of the feature map, generating too many anchors not only reduces the detection accuracy but also brings computational complexity increase.

Different form the RPN in Faster R-CNN, whose box dimension are hand-picked, the anchors generated are based on the average size of the foreground target, which can make it easier for the regression network to learn to predict good locations. As shown in Figure 5.

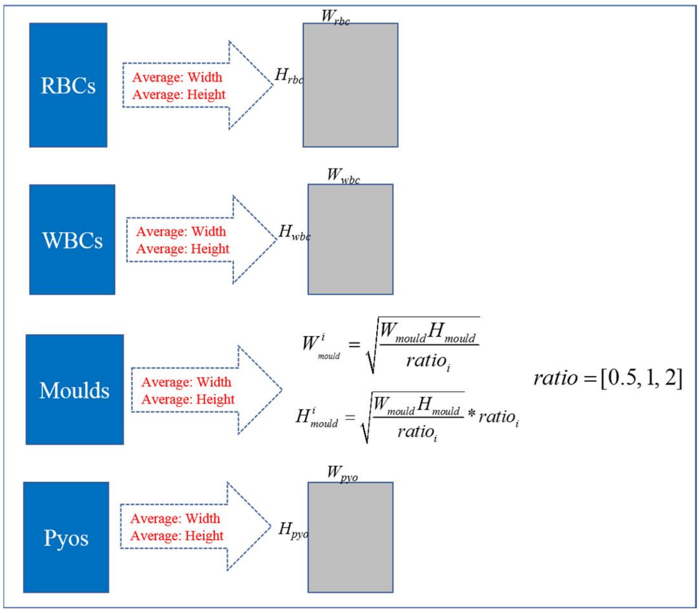

Figure 5. Generation of anchors

The average IOU to closest prior of our producing strategy and the Faster R-CNN strategy are compared in Table 1. There are 6 priors, which performs better to the anchors in Faster R-CNN.

Table 1. Average IOU of anchors to closest priors on training set

**Anchors generation**	**#**	**Avg IOU**
Our strategy	6	71.6
Faster R-CNN	9	67.0

3.1.3 PCA based Classification and Box Regression

After generating the region proposal box, a classification and regression head is need to classify and locate. Because the cell volume is smaller in whole image field,

the ROI-Pooling strategy in Faster-RCNN has no obvious effect on feature aggregation. The position sensitive mapping strategy in R-FCN[21] is based on the ROI-Pooling strategy, which has the same AP of the target detection to Faster R-CNN. In this paper, we adopt the PCA strategy to replace ROI-Pooling strategy, which achieve the higher AP (PCA-Faster-RCNN).

In general, the average size of the RBC in fecal images is about 48*48, WBC 58*58, and the size range of the mildew varies from 30*30-108*108. Thus, the rectangle size of the extracted region proposal in feature map ranged from 2*2 to 7*7. In order to achieve the consistency of the feature dimensions of all the candidate regions, PCA strategy is used to reduce to the same dimension.

We transform the feature maps extracted from the training set to the 1*1*N dimensional feature vectors. For each feature vector, PCA method is used to reduce it to 1024 dimensions. It is found that the feature of the candidate set can be preserved to the maximum extent, and the retention rate is more than 97% after the experiments.

Each fixed-length feature vector is input into several full connected layers and the final feature vector is fed into two layers: (1) Softmax layer produces 5 types of probability scores (4 types of cells plus background) and (2) the regression layer for predicting the relative coordinates.

3.2 Training process

3.2.1 RPN Training

Before RPN training, each region proposal should be labeled as foreground or background to indicate whether an object is contained in the region or not. If the intersection over union (IOU) of a box which produced by an anchor who can map to the original image and the ground-truth box on the origin is greater than 0.7, the box produced by the anchor is the foreground, otherwise, and the anchors with IOU less than 0.1 are the background. The loss function of RPN can be written as:

$$L(s,t_{x,y,w,h}) = \frac{\lambda_{cls}}{N_{cls}} \sum_i L_{cls}(c_i, c_i^*) + \frac{\lambda_{reg}}{N_{reg}} \sum_i [c_i^* > 0] L_{reg}(t_i, t_i^*) \quad (1)$$

where λ_{cls} is the weights for classification loss, while λ_{reg} is the weights for box regression. c_i* is the ground-truth label (background/foreground) of the region proposed, t_i is a vector representing the 4 parameterized coordinates of the predicted bounding box, and t_i* is that of the ground-truth box associated with a positive anchor.

The first item $L_{cls}(c_i, c_i^*)$ is the classification loss (cross-entropy loss), and $L_{reg}(t_i, t_i^*)$ is the box regression loss (smooth $L1$ loss[14]). $[c_i^*>0]$ represents for the positive samples (foreground). N_{cls} and N_{reg} are the normalization factor.

When training, not all anchors in RPN is used for training. Generally, anchor with the same amount of foreground and background is selected by random sampling to train.

During the test, the foreground target may be detected by many anchors, each anchor has a corresponding score, that is, the probability (confidence) classified as the foreground target. Duplicate anchors need to be filtered out. Non-maximum suppression (NMS) was used as the filtering method. In addition, some anchors in the original image are far beyond the image boundary, which also need to be filtered. The final output process of RPN is summarized as follows:

1. Generate anchors, and then calculate the scores of each anchor through RPN network.
2. Map the anchor to the original image to determine whether the anchor has gone beyond the boundary in a wide range, and if so, remove the anchor.
3. Ranking the score of foreground anchor from large to small, extracting the first N anchors, that is, the N anchors are foreground anchors after position correction;
4. NMS arithmetic is applied to anchor in step 3.
5. According to the ranking of anchor after NMS algorithm, the first M anchors are extracted as the foreground output of RPN.

Experiments show that when M is 2000 or 300, it has little effect on the final detection. Therefore, to simplify the calculation, 300 is more suitable.

3.2.2 Model Training

The training adopts the method of training the RPN module jointly with the object recognition network, rather than alternating. Since the structure of the Faster R-CNN is end-to-end, both the RPN and the object recognition network can provide feedback on the feature extraction layer. During the backpropagation, loss from both the RPN and the fast R-CNN are combined and calculated together.

We introduced PCA strategy in the classification and regression part of Faster R-CNN, which should be trained separately. An origin Faster R-CNN model is created as M_0, who has the improved RPN network (3.1.2) and the ROI pooling strategy. PCA based Faster R-CNN is marked as M_1. The training process can be shown in Figure 6.

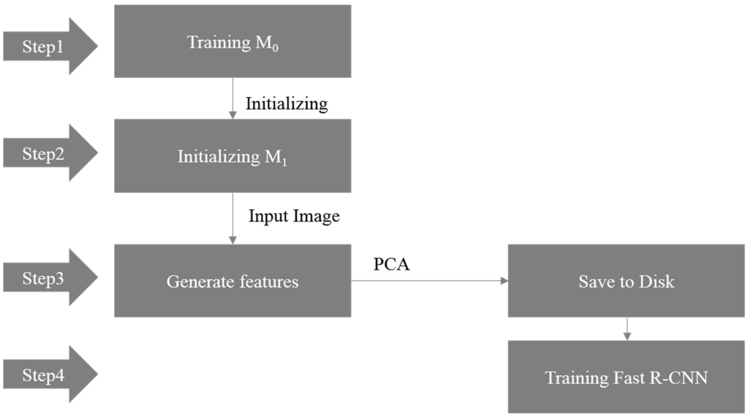

Figure 6. Diagram of training process

References

1 Ballard, H. & Brown, C. M. *Computer Vision*, <http://www.bmva.org/visionoverview> (1982).

2 Gautam, A., Singh, P., Raman, B., Bhadauria, H. & Ieee. *Automatic Classification of Leukocytes using Morphological Features and Naive Bayes Classifier*. (2016).

3 Liu, L. *et al.* Automatic identification of fungi under complex microscopic fecal images. *Journal of Biomedical Optics* **20**, doi:10.1117/1.Jbo.20.7.076004 (2015).

4 Piuri, V. & Scotti, F. in *2004 IEEE International Conference onComputational Intelligence for Measurement Systems and Applications, 2004. CIMSA.* 103-108.

5 Wang, M. *et al.* Novel cell segmentation and online SVM for cell cycle phase identification in automated microscopy. *Bioinformatics* **24**, 94-101, doi:10.1093/bioinformatics/btm530 (2008).

6 Manik, S., Saini, L. M. & Vadera, N. in *2016 IEEE 1st International Conference on Power Electronics, Intelligent Control and Energy Systems (ICPEICES).* 1-5.

7 Ghosh, P., Bhattacharjee, D. & Nasipuri, M. Blood smear analyzer for white blood cell counting: A hybrid microscopic image analyzing technique. *Appl Soft Comput* **46**, 629-638 (2016).

8 Kim, E., Cortre-Real, M. & Baloch, Z. in *Medical Imaging 2016: Pacs and Imaging Informatics: Next Generation and Innovations* Vol. 9789 *Proceedings of SPIE* (eds J. Zhang & T. S. Cook) (2016).

9 Afridi, M. J. *et al.* Intelligent and Automatic In Vivo Detection and Quantification of Transplanted Cells in MRI. *Magn Reson Med* **78**, 1991-2002 (2017).

10 Zhang, J. *et al.* Computerized detection of leukocytes in microscopic leukorrhea images. *Med Phys* **44**, 4620-4629 (2017).

11 Zhang, J. *et al.* Automatic identification of fungi in microscopic leucorrhea images. *Journal of the Optical Society of America a-Optics Image Science and Vision* **34**, 1484-1489, doi:10.1364/josaa.34.001484 (2017).

12 Girshick, R., Donahue, J., Darrell, T. & Malik, J. in *2014 IEEE Conference on Computer Vision and Pattern Recognition.* 580-587.

13 Girshick, R. in *2015 IEEE International Conference on Computer Vision (ICCV).* 1440-1448.

14 Ren, S., He, K., Girshick, R. & Sun, J. Faster R-CNN: Towards Real-Time Object Detection with Region Proposal Networks. *IEEE Transactions on Pattern Analysis and Machine Intelligence* **39**, 1137-1149, doi:10.1109/TPAMI.2016.2577031 (2017).

15 Zhang, J. K., Hu, H. G., Chen, S. Y., Huang, Y. J. & Guan, Q. Cancer Cells Detection in Phase-Contrast Microscopy Images based on Faster R-CNN. *Int Sym Comput Intel*, 363-367, doi:10.1109/Iscid.2016.89 (2016).

16 Hung, J. *et al.* in *IEEE Conference on Computer Vision and Pattern Recognition Workshops* 2160-2174 (Honolulu, Hawaii, USA, 2017).

17 Kang, R., Liang, Y., Lian, C. & Mao, Y. CNN-Based Automatic Urinary Particles Recognition. (2018).

18 *ImageNet Large Scale Visual Recognition Competition 2014 (ILSVRC2014)*, <http://image-net.org/challenges/LSVRC/2014/index> (2014).

19 He, K., Zhang, X., Ren, S. & Sun, J. in *29th IEEE Conference on Computer Vision and Pattern Recognition(CVPR).* 770-778 (IEEE Computer Society).

20 *COCO: Common Object in Context*, < https://cocodataset.org/> (2015).

21 Dai, J., Li, Y., He, K. & Sun, J. in *Proceedings of the 30th International Conference on Neural Information Processing Systems* 379–387 (Curran Associates Inc., Barcelona, Spain, 2016).
